# Can fluid responsiveness tests utilizing positive end-expiratory pressure changes be adapted to improve applicability in all mechanically ventilated patients?

**DOI:** 10.1186/s13054-023-04483-w

**Published:** 2023-05-17

**Authors:** Camilo Pérez, Laura Castillo, Jorge Alvarado

**Affiliations:** 1 Critical and Intensive Care Medicine Department, Hospital Universitario Fundación Santa Fe de Bogotá, Bogotá, Colombia; 2 School of Medicine and Health Sciences, Universidad del Rosario, Bogotá, Colombia; 3 Department of Physiology, National University of Colombia, Bogotá, Colombia

We were enthusiastic about reading the article by Lai et al. which cleverly predicts fluid responsiveness in low tidal volume ventilated patients by utilizing changes in positive end-expiratory pressure (PEEP) on cardiac output [1]. We applaud the authors for demonstrating once again the effect of PEEP on the performance of fluid responsiveness tests.

However, we want to highlight certain limitations of the study, such as the inclusion of only low tidal volume ventilated patients with PEEP greater than 10 cmH2O, who mostly presented low respiratory system compliance. This narrows down the applicability of the results and adds to the long list of variables that affect fluid responsiveness tests, such as spontaneous efforts, tidal volume, lung and chest wall elastance, recruited volume, cardiac function, and heart rate, among others [2]. Although the study results are positive, it is still unclear whether a test based on these principles can be useful for all mechanically ventilated patients. It is likely that this is not possible, prompting us to question the possibility of adjusting the cutoff points of these tests based on the aforementioned variables to make them useful for the majority of patients.

If we assume that changes in stroke volume during the respiratory cycle are caused by changes in transpulmonary pressure (PL) and pleural pressure (Ppl) [2, 3], we can draw some conclusions. Previous research has shown that changes in transpulmonary pressure related to alterations in positive end-expiratory pressure (PEEP) are proportional to increases in PEEP [4]. Therefore:

$$\Delta {\text{PL}} = \Delta {\text{PEEP}}$$

$$\Delta {\text{PL}} = {\text{PEEP}}_{{{\text{High}}}} - {\text{PEEP}}_{{{\text{Low}}}}$$

Assuming that transpulmonary pressure (PL) is the difference between plateau pressure (Paw) and pleural pressure (Ppl) (PL = Paw − Ppl) and that driving pressure (DP) is the difference between Paw and PEEP (DP = Paw − PEEP), we can draw some conclusions:

$$\Delta {\text{PL}} = \Delta {\text{Paw}} - \Delta {\text{Ppl}}$$

$$\Delta {\text{Ppl}} = \Delta {\text{Paw}} - \Delta {\text{PL}}$$

$$\Delta {\text{Ppl}} = \Delta {\text{Paw}} - \Delta {\text{PEEP}}$$

$$\Delta {\text{Ppl}} = \left( {{\text{Paw}}_{{{\text{High}}}} - {\text{Paw}}_{{{\text{Low}}}} } \right) - \left( {{\text{PEEP}}_{{{\text{High}}}} - {\text{PEEP}}_{{{\text{Low}}}} } \right)$$

$$\Delta {\text{Ppl}} = \left( {{\text{Paw}}_{{{\text{High}}}} - {\text{PEEP}}_{{{\text{High}}}} } \right) - \left( {{\text{Paw}}_{{{\text{Low}}}} - {\text{PEEP}}_{{{\text{Low}}}} } \right)$$

$$\Delta {\text{Ppl}} = {\text{DP}}_{{{\text{High}}}} - {\text{DP}}_{{{\text{Low}}}}$$

$$\Delta {\text{Ppl}} = \Delta {\text{DP}}$$

Although the study did not find a correlation between the recruitment-to-inflation ratio and the hemodynamic changes after the PEEP test, it is important to note that this ratio is not a direct measure of the change in lung volume at end-expiration but rather evaluates the probability of recruitment [5]. Additionally, the study did not have sufficient statistical power to test the correlation between the magnitude of the increase in the cardiac index and the change in PEEP. Therefore, it is possible that there is a correlation that was not detected by the study due to its limited statistical power.

To summarize, the cutoff point for changes in the cardiac index after the PEEP test can only predict fluid responsiveness in a limited group of patients. There is still room for exploration in terms of adjusting these cutoff points based on the mechanics of the respiratory system. One potential proposal is to base it on changes in PEEP, end-expiratory lung volume, and driving pressure, but this requires further demonstration.

## Data Availability

Not applicable.

## Abbreviations

DP: Driving pressure
Paw: Plateau pressure
PEEP: Positive end-expiratory pressure
PL: Transpulmonary pressure
Ppl: Pleural pressure
